# Protective Effects of Ozone against Quinolinic Acid-Induced Redox Imbalance in Murine BV-2 Microglial Cells

**DOI:** 10.1007/s12640-026-00798-y

**Published:** 2026-04-16

**Authors:** Pedro Henrique Zatti, Nicole Peyrot da Silva, Marina Rigotti, Fernando Joel Scariot, Carolina Bordin Davidson, Alencar Kolinski Machado, Catia Santos Branco

**Affiliations:** 1 Laboratory of Oxidative Stress and Antioxidants, Biotechnology Institute, University of Caxias do Sul, Caxias do Sul, RS Brazil; 2 Laboratory of Enology and Applied Microbiology, Biotechnology Institute, University of Caxias do Sul, Caxias do Sul, RS Brazil; 3 Cell Culture & Bioactive Effects Laboratory, Franciscan University, Santa Maria, RS Brazil

**Keywords:** Ozone therapy, Kynurenine pathway, Neuroglia, Neurodegeneration

## Abstract

Ozone gas (O₃) has emerged as a promising therapeutic agent for various pathological conditions, including neurodegenerative diseases (NDs). These illnesses are associated with the accumulation of neurotoxic metabolites by microglia, including tryptophan catabolites. This study aimed to investigate the effects of O₃ exposure on BV-2 microglial cells in the presence or absence of quinolinic acid (QA). O₃ at increasing concentrations (5, 12, 20, 40, and 70 µg/mL) was added in cell culture medium, both under baseline conditions and following exposure to QA (1.5 mM) for 24 h. Spectrophotometry and flow cytometry were performed to assess cell viability, apoptosis, reactive oxygen species (ROS), nitric oxide (NO), mitochondrial membrane potential (ΔΨm), lipid peroxidation (TBARS), and the activities of superoxide dismutase (SOD) and cytochrome *c* oxidase (COX). qRT-PCR assessed TNF-α gene expression levels. O₃ exposure enhanced cell viability without inducing oxidative/nitrosative stress or DNA damage in BV-2 cells. In addition, 12 µg/mL of O₃ significantly improved cell viability and alleviated oxidative stress and inflammation induced by QA by normalizing SOD activity, reducing lipid peroxidation, and TNF-α gene expression. Nevertheless, O₃ did not restore COX activity nor prevent the hyperpolarization observed under co-treatment conditions. The results suggest that O₃ exerts a biphasic effect, depending on the cellular redox state. This study provides critical insights into the complex mechanisms underlying ozone action in glial cells, highlighting its potential for developing new therapeutic strategies for NDs.

**Graphical Abstract:**

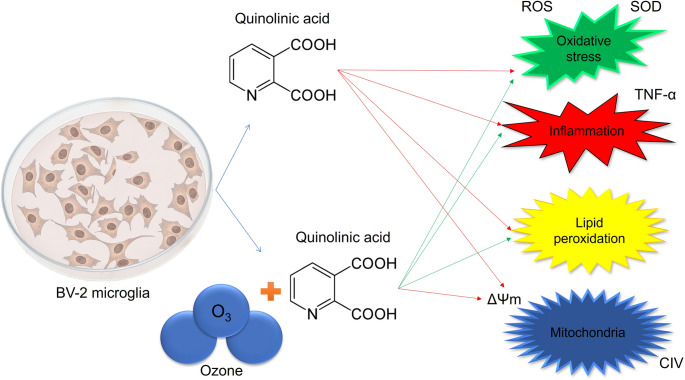

**Supplementary Information:**

The online version contains supplementary material available at 10.1007/s12640-026-00798-y.

## Introduction

Neurodegenerative diseases (NDs), such as Alzheimer’s and Parkinson’s disease, and multiple sclerosis, represent a growing global public health challenge, characterized by the progressive loss of neuronal structure and function (Erkkinen et al. 2018; Galts et al. 2019). A central and consistent denominator associated with these conditions is chronic neuroinflammation, a state of sustained cellular activation by glial cells that precedes or accompanies neural damage. Microglia, the central nervous system’s resident immune cells, play a crucial role in this process (Tang and Le 2016; Dugger and Dickson 2017; Prinz and Priller 2017). In a healthy state, microglia act as sentinels and phagocytes, adopting a protective phenotype (M2). However, in pathological environments, they polarize toward a pro-inflammatory phenotype (M1), a state characterized by the massive release of cytotoxic mediators, including reactive oxygen species (ROS), nitric oxide (NO), and pro-inflammatory cytokines such as Tumor Necrosis Factor-alpha (TNF-α) (Wu et al. 2023). It is this persistent microglial dysfunction, accompanied by neuronal loss and subsequent release of toxic catabolites, in which the connection between inflammation and the neurotoxicity observed in NDs is established.

In this scenario of inflammation and toxicity, the kynurenine pathway (KP), the main metabolic route for tryptophan, becomes critically dysregulated (Salminen 2022). Under pathological conditions, the enzymatic activity of this pathway is altered, shifting metabolism toward the formation of neurotoxic metabolites. The most prominent and potent of these is quinolinic acid (QA) (Davidson et al. 2022). QA acts as an endogenous agonist of N-methyl-D-aspartate (NMDA) receptors, and its accumulation is directly associated with excitotoxicity, oxidative stress, and mitochondrial dysfunction in both neurons and glial cells (Török et al. 2020; Liang et al. 2022). Therefore, QA is frequently utilized in in vitro and in vivo models to mimic neuroinflammation, as it induces redox imbalance, mitochondrial dysfunction, and an inflammatory response in neurons and glial cells (Pierozan et al. 2018; Stepanova et al. 2020; Abbasinezhad-Moud et al. 2023; Ge et al. 2023; Amiri et al. 2024; Gandhi and Panchal 2025; Jiang et al. 2025; Rigotti et al. 2025; Santos et al. 2025).

In the search for therapeutic strategies that can modulate this neurotoxic cascade, ozone therapy emerges as a promising alternative. This approach involves the controlled application of an oxygen-ozone (O_2_/O_3_) gas mixture, whose action is related to its ability to induce a beneficial and adaptive response through its high oxidative capacity (Di Mauro et al. 2019). Crucially, the biological response is concentration-dependent: while ozone can be toxic at high concentrations, low and controlled concentrations act as signaling inducers (Borges et al. 2017). These low doses promote a transient oxidative stress that, in turn, activates endogenous antioxidant systems (such as the Nrf2 pathway), resulting in the modulation of oxidative stress, ROS production, and inflammation (cytokine production) across various models (Zavala et al. 2016; Poma et al. 2017; Costanzo et al. 2020; Tang et al. 2021; Li et al. 2021; Bowers et al. 2021; Cisterna et al. 2021; Santos et al. 2021; Orlandin et al. 2022; Carmora et al. 2025). In the context of NDs, ozone therapy has demonstrated potential in modulating markers of oxidative stress, cellular metabolites, as well as promoting cell viability and reducing neural degeneration in vivo (Delgado-Roche et al. 2017; Bette et al. 2022). Despite these promising results, the literature lacks detailed mechanistic data on how O_3_ interacts with and reverses neurotoxicity induced by metabolites like QA.

To bridge this knowledge gap, the present study investigated the effects of ozone on quinolinic acid-induced toxicity. Thus, the objective of this work was to elucidate how ozone exposure can reverse the redox imbalance and microglial inflammation induced by quinolinic acid. This provides insights into the therapeutic potential of O_3_ as an agent capable of modulating the pathophysiological mechanisms associated with the kynurenine pathway in neurodegenerative diseases.

## Materials and Methods

### Cell Culture

BV-2 microglial cell line was acquired from the Rio de Janeiro Cell Bank (BCRJ). BV-2 cells were cultivated in RPMI medium supplemented with 10% (v/v) fetal bovine serum (FBS) and 1% (v/v) penicillin–streptomycin. The cells were maintained in culture flasks at 37 °C within a humidified atmosphere containing 5% CO₂. Daily assessments were performed via microscopy to monitor growth conditions. The culture medium was replenished as required until the cells achieved 80% to 90% confluence, which represents the optimal stage for subsequent experimental procedures.

### O_3_ Effects in a Non-Stress Environment

**Fig. 1:**
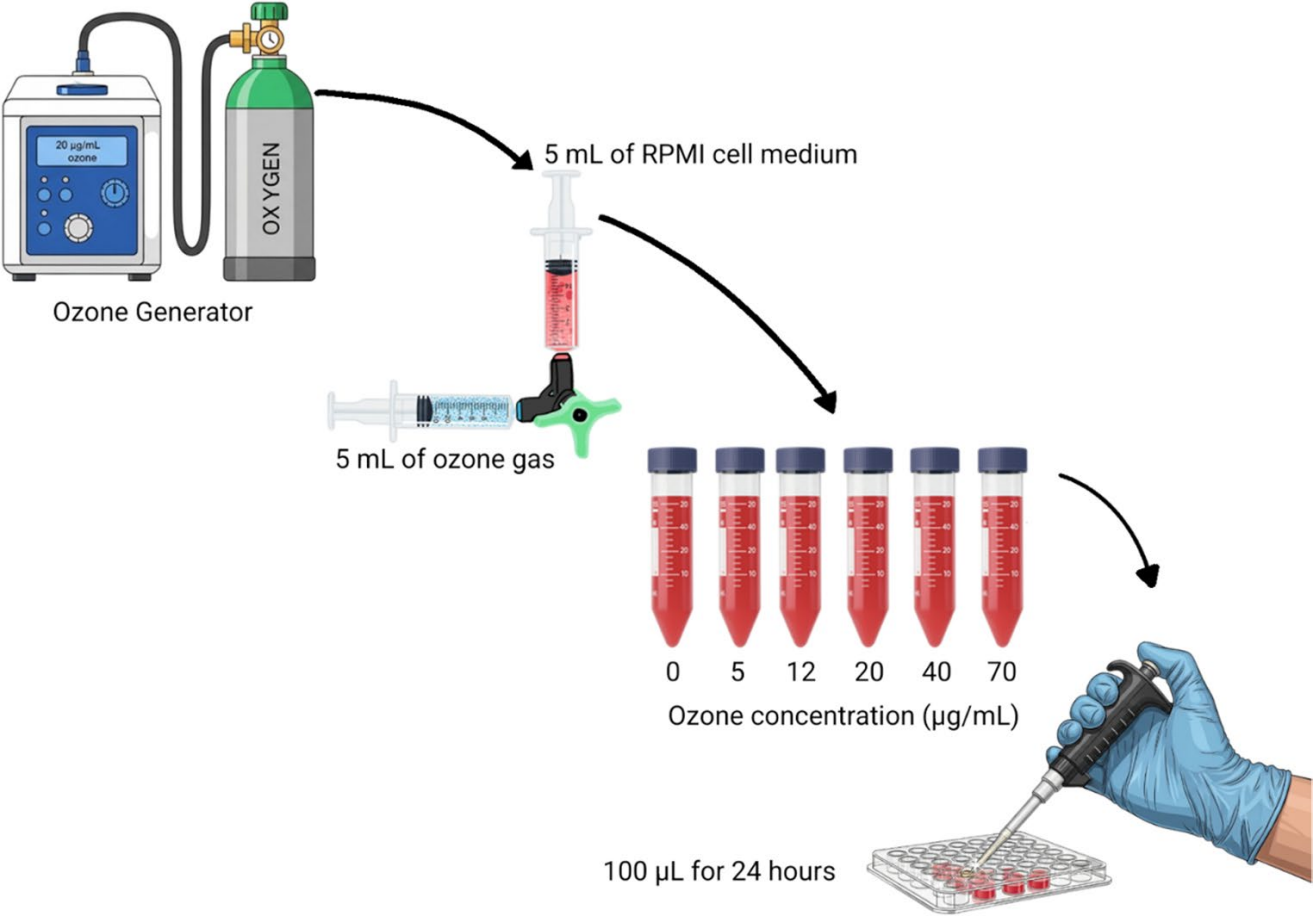
Method design of cellular ozone exposure

Ozone gas was produced using an ozone generator (Pro3 Medical, São Paulo, Brazil). It was solubilized in the culture medium at increasing concentrations (5, 12, 20, 40, and 70 µg/mL) for 5 min, with an oxygen flow of 3⁄4 L/min (Fig. 1). These concentrations are equivalent to doses usually administered in clinical practice and described in previous in vitro studies (Borges et al. 2017; Li et al. 2021; Cisterna et al. 2021). The contact time of the cells with the ozonized medium was 24 h. Negative control consisted of cells without O_3_ exposure. After the determined time, analyses were performed to determine cell viability, nitric oxide levels, ROS production, and nuclear membrane integrity. Results were expressed as a percentage of the control.

#### Cell Viability

To evaluate cell viability, a 3-(4,5-dimethylthiazol-2yl-)-2,5-diphenyl tetrazolium bromide (MTT) assay was performed, according to the methodology of Mosmann (1983), with modifications. First, 1 × 10^5^ cells were seeded in 96-well plates and allowed to grow for 24 h. After this period, the culture medium was replaced, and treatments were immediately added. Cells were treated with 100 µL of fresh medium (negative control) or 100 µL of ozonized medium (prepared immediately prior to its application) for 24 h. The ozonized medium remained in contact with the cells for the entire 24-hour period. Following the treatment period, the supernatant was carefully collected from the wells for subsequent biochemical and molecular analyses (e.g., dsDNA, NO, ROS). Then, the remaining treatment medium was removed, and the MTT stock solution (1 mg/mL) was diluted in serum-free medium (1:3) and added to wells. Plates were incubated for three hours at 37 °C. Subsequently, the MTT solution was removed, and the plate was left to dry for 24 h. Formazan violet crystals were dissolved with 100 µL of dimethylsulfoxide (DMSO), stirred for 15 min, and the absorbance was read at 570 nm using a microplate reader (Victor-X3, Perkin Elmer, Finland). The assay was repeated in at least three independent biological experiments (*n* ≥ 3). The results were expressed as a percentage of the control.

#### Nitric Oxide (NO) Levels

Cellular nitrosative stress was evaluated by monitoring NO levels, an important inflammatory mediator. The analysis was conducted using the Griess reagent. Nitrite (NO₂⁻) metabolite concentrations were determined based on a sodium nitroprusside standard curve, as described by Green et al. (1981). After 24 h of treatment as previously described, the extracellular medium (50 µL of supernatant) from each well was collected and transferred to a new 96-well plate. Then, 50 µL of Griess reagent was added to the wells containing the medium. The plate was then incubated at room temperature for 15 min. The absorbance was read at 550 nm (Victor-X3, Perkin Elmer, Finland). The assay was repeated in at least three independent biological experiments (*n* ≥ 3). Results were expressed as a percentage of the control.

#### Reactive Oxygen Species (ROS) Levels

To evaluate if ozone exposure induces oxidative stress, ROS levels were quantified utilizing the 2,7-dichlorofluorescein diacetate fluorometric assay, as detailed by Degli Esposti (2002). For this assay, 1 × 10^5^ cells were seeded per well in a 96-well plate and treated according to the experimental design. This methodology relies on the conversion of dichlorofluorescein (DCFH-DA) to dichorodihydrofluorescein (DCFH) via deacetylation, which is subsequently oxidized by reactive species into dichlorofluorescein (DCF). Measurements were performed using a fluorescence reader (Victor-X3, Perkin Elmer, Finland), with excitation at 485 nm and emission at 525 nm. The assay was repeated in at least three independent biological experiments (*n* ≥ 3). Results were expressed as a percentage of the control.

#### Integrity of the Nuclear Membrane

The concentration of extracellular double-stranded DNA (ds-DNA) was determined using the Quant-iT™ PicoGreen^®^ dsDNA dye (Invitrogen Life Technologies). PicoGreen is a sensitive fluorescent reagent that binds to dsDNA molecules, enabling the assessment of cellular integrity. The sample consisted of 10 µL of the culture supernatant previously collected after the 24-hour treatment period. For the assay, 80 µL of 1X TE buffer was dispensed into a 96-well plate, followed by the addition of 10 µL of the sample. Subsequently, 10 µL of PicoGreen^®^ reagent was added, and the mixture was incubated at room temperature for 5 min, as per the methodology described by Ha et al. (2011). Fluorescence was measured using a Victor-X3 fluorescence reader (Perkin Elmer, Finland) with excitation at 480 nm and emission at 520 nm. The assay was repeated in at least three independent biological experiments (*n* ≥ 3). Results were normalized and expressed as a percentage of the control.

### O_3_ Effects in a Stressful Environment

To investigate the microglial response to stress stimuli, BV-2 cells were exposed to quinolinic acid (QA) at a concentration of 1.5 mM for 24 h. Initially, two concentrations of QA (1.5 mM and 2.0 mM) were tested, and comparative data are available in the Supplementary Material (SM1). As both concentrations demonstrated similar levels of cellular toxicity and redox imbalance, the lower concentration (1.5 mM) was selected, also grounded by our previous study data (Rigotti et al. 2025). The experimental design included groups treated with QA, both in the presence and absence of ozone. After treatments, cell viability, NO and ROS levels, and the extracellular concentration of dsDNA were analyzed, as detailed in Sect.  2.2.1 to 2.2.4.

### Molecular Analysis by Flow Cytometry

Assays were performed with an inoculum of 2 × 10^6^ cells treated with experimental compounds (QA, O_3_, or QA + O_3_) for 24 h before analysis. The fluorescence intensity of 10,000 cells was quantified using a BD FACSCalibur four-laser flow cytometer (Becton Dickinson Ltd). Data acquisition was conducted with CellQuest Pro software (BD Biosciences), and subsequent analysis was executed using FlowJo (TreeStar, Inc.). All flow cytometry experiments were performed in at least two independent biological experiments.

#### Apoptosis

Apoptosis was assessed by quantifying phosphatidylserine levels on the outer cell membranes using the Invitrogen™ Annexin V-FITC/PI apoptosis detection kit. Following the 24-hour treatment period, cells were washed with PBS, resuspended in Annexin V binding buffer, and stained with Annexin V-FITC and Propidium Iodide (PI) for 15 min at room temperature, according to the manufacturer’s instructions. Results were expressed as a percentage of the control.

#### Mitochondrial Membrane Potential (ΔΨm)

Following the 24-hour treatment period, cells were resuspended in PBS (pH 7.4) and stained with 175 nM of 3,30-dihexyloxacarbocyanine iodide (DiOC6) for 30 min at 30 °C. Subsequently, cells were analyzed on a FL1 filter using flow cytometry (FACSCalibur instrument; Becton-Dickinson). A total of 10,000 cells were analyzed for each sample, and the results were expressed as a percentage of the control.

### Enzymatic Activities

For all enzymatic assays, cells were treated with experimental compounds (AQ, O_3,_ or AQ + O_3_) for 24 h before lysis. Then, cell lysates were prepared, and protein concentration was determined before the assay. All enzymatic assays were repeated in at least three independent biological experiments (*n* ≥ 3).

#### Superoxide Dismutase (SOD)

SOD activity was quantified to evaluate endogenous antioxidant levels, employing the method described by Bannister and Calabrese (1987). Lysates were homogenized in glycine buffer (50 mmol, pH 10.2), pre-incubated for 2 min at 30 °C, after which epinephrine was introduced to initiate the reaction. The kinetic progression was monitored spectrophotometrically at 480 nm for 3 min. Results were presented as SOD units per milligram of protein. One unit of SOD activity is defined as the quantity required to inhibit the rate of epinephrine oxidation by 50%.

#### Cytochrome *c* Oxidase (COX)

COX activity, representing mitochondrial complex IV within the electron transport chain (ETC), was assessed following the methodology described by Spinazzi et al. (2012). Cell lysates were prepared after the 24-hour treatment period, and the protein content was quantified for normalization. Measurements were performed at a wavelength of 550 nm over three minutes at 37 °C, using potassium cyanide as a specific inhibitor. Kinetic readings were monitored spectrophotometrically using a Genesys™ 10 S UV-VIS spectrophotometer (Thermo Scientific, USA), and the data were presented as a percentage relative to the control group.

### Lipid Peroxidation

Lipid peroxidation was assessed by quantifying Thiobarbituric Acid Reactive Substances (TBARS) in cell lysate. Peroxidation products were measured using the thiobarbituric acid colorimetric reaction for malondialdehyde (MDA), as described by Wills (1966). Cell lysates were prepared after the 24-hour treatment period, and the protein content was quantified for normalization. Tetraethoxypropane was employed for calibration, a compound that quantitatively produces the MDA-thiobarbituric acid adduct. Results were reported in nM/mg protein.

### Tumor Necrosis Factor alpha (TNF-α) Expression

This assay followed the protocol described by Davidson et al. (2024). RNA was extracted using TRI Reagent^®^ (Sigma-Aldrich, Saint Louis, MO, USA) using an in-house methodology and quantified using the NanoDrop Lite^®^ (ThermoFisher Scientific, Wilmington, DE, USA) equipment. Complementary DNA (cDNA) was produced using the iScript™ cDNA Synthesis kit (Bio-Rad, Hercules, CA, USA), and the amount of RNA was normalized for each sample to a final concentration of 500 ng. Quantitative real-time PCR was carried out using a GoTaq^®^ qPCR Master kit (Promega, Madinson, WI, USA). qRT-PCR cycles were as follows: (1) 50°C for 120 s; (2) 95°C for 120 s; and (3) 40 cycles of 95°C for 15 s followed by 60°C for 30 min. TNF-α primers were as follows: forward − 5’ TTGCTCTGTGAAGGGAATGG 3’; reverse − 5’ GCTCTGAGGAGTAGACAATAAAG 3’. GAPDH was the housekeeping gene. GAPDH primers were as follows: forward − 5’ AGTCAGCCGCATCTTCTTTT 3’; reverse − 5’ ACCAGAGTTAAAAGCAGCCC 3’. Gene expression data were analyzed using the delta–delta Ct method and converted to relative expression levels, which were then compared to the control group. qRT-PCR experiments were performed in at least two independent biological experiments.

### Statistical Analysis

All assays were conducted with independent biological replicates on separate days, each including technical replicates. Results are presented as mean ± standard deviation (SD). Statistical analysis was performed using GraphPad Prism version 9 for Windows (Boston, Massachusetts, USA). Data was analyzed via one-way analysis of variance (ANOVA) for ozone individual effects, and two-way ANOVA followed by Tukey’s *post hoc* test to investigate interactions between treatments. A significance level of *p* ≤ 0.05 was adopted.

## Results

In the present study, BV-2 murine microglial cells were used to investigate the effects of ozone in the presence or absence of QA, a downstream product of tryptophan metabolism. This cell line is usually employed in neuroinflammation research (Avallone et al. 2020; Janpaijit et al. 2023; Sheu et al. 2023; Bayurova et al. 2023).

### O_3_ Effects in a Non-Stress Environment

The initial phase of this study focused on developing a robust and reliable ozonation method for treating culture media. Two distinct systems were evaluated: a closed system utilizing syringes for ozone homogenization and an open system employing glass tubes. The closed system proved superior due to its ability to precisely regulate the injected gas volume, ensuring consistent and reproducible ozone concentrations. Conversely, the open system exhibited significant limitations in gas volume control, thereby compromising its reliability and precluding its validation for subsequent experiments (data not shown). The precision offered by the syringe-based, closed system was crucial for ensuring accurate and repeatable experimental conditions.

**Fig. 2:**
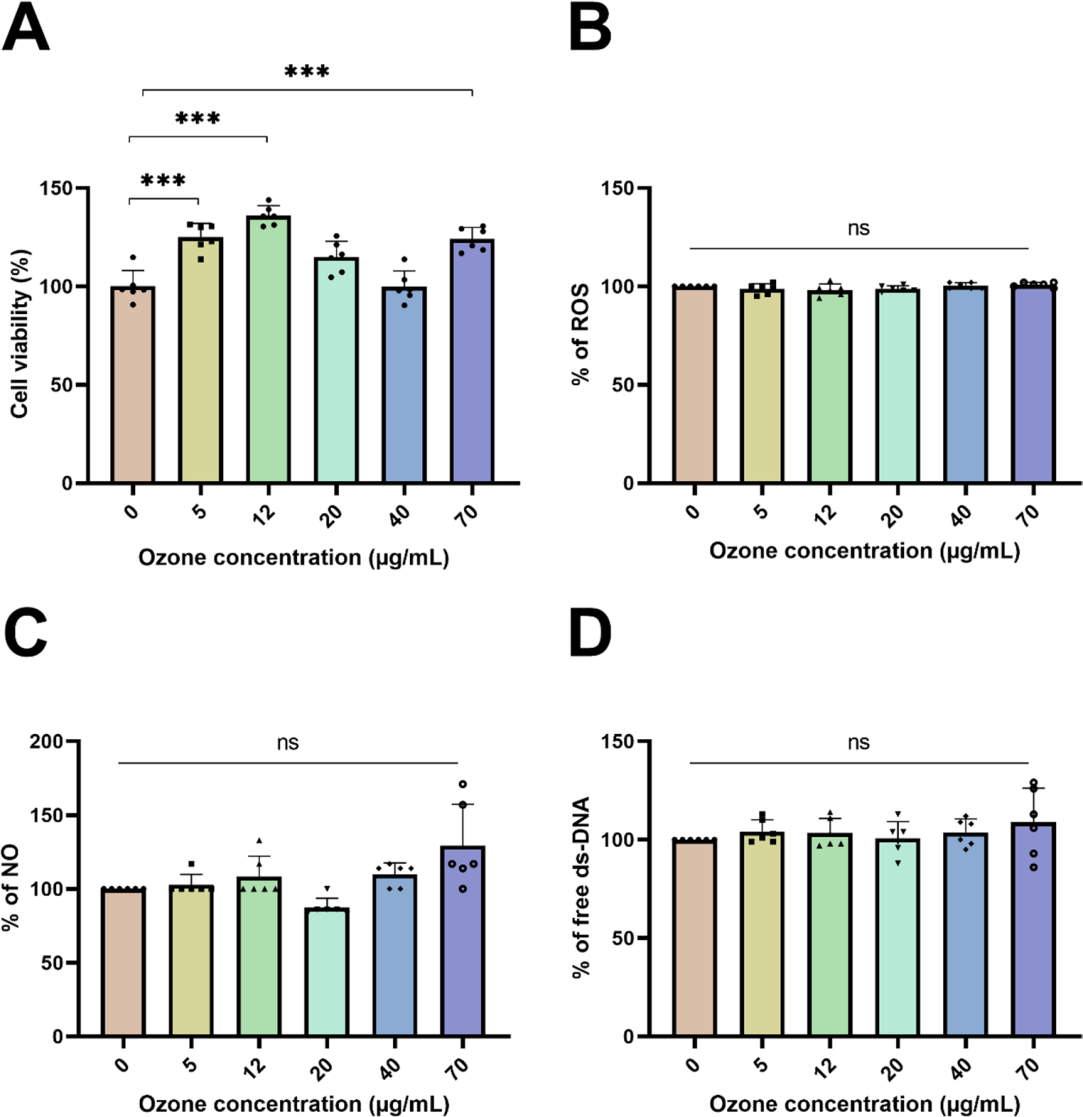
The effects of ozone on murine microglial (BV-2) cell viability (**A**), reactive oxygen species levels (**B**), nitric oxide production (**C**), and quantification of free ds-DNA (**D**) in cells treated with increasing ozone concentrations (0, 5, 12, 20, 40, and 70 µg/mL) for 24 h. Results are expressed as mean ± standard deviation, with individual data points shown in each parameter (six experimental replicates and are representative of at least three independent experiments; *n* = 6). Statistically significant differences were determined by one-way ANOVA with multiple comparisons between the control and the ozone concentrations and indicated by asterisks (*p < 0.05; ** *p* < 0.01; *** *p* < 0.001; **** *p* < 0.0001). Statistical significance was set at *p* ≤ 0.05. QA: Quinolinic Acid; O_3_: Ozone

Following 24 h of ozonation, a comprehensive analysis of cell viability across various ozone concentrations was conducted (Fig. 2A). The results demonstrated a clear concentration-dependent effect, with an optimal viability observed at a concentration of 12 µg/mL (136 ± 5%; *p* < 0.001) compared to the untreated control group. Cell viability was also significantly increased at 5 µg/mL (125 ± 7%; *p* < 0.001) and 70 µg/mL (124 ± 6%; *p* < 0.001). This finding highlights the potential of ozonation to positively influence cellular metabolism under an appropriate concentration window.

To evaluate whether alterations in cell viability were linked to nitrosative and oxidative stress, relevant markers were assessed. Analysis of Reactive Oxygen Species (ROS) levels (Fig. 2B). Nitric Oxide (NO) levels (Fig. 2C), and quantification of ds-DNA release (Fig. 2D) did not reveal any statistically significant alteration across all tested ozone concentrations compared to the control group. This strongly suggests that O_3_ exposure, within the tested range, does not overtly induce oxidative stress or compromise nuclear integrity.

Considering all results, 12 µg/mL was selected as the optimal concentration for subsequent experiments because it provided the highest statistically significant increase in cell viability without inducing detectable oxidative or nitrosative stress markers.

### O_3_ Effects in a Stress Environment

**Fig. 3:**
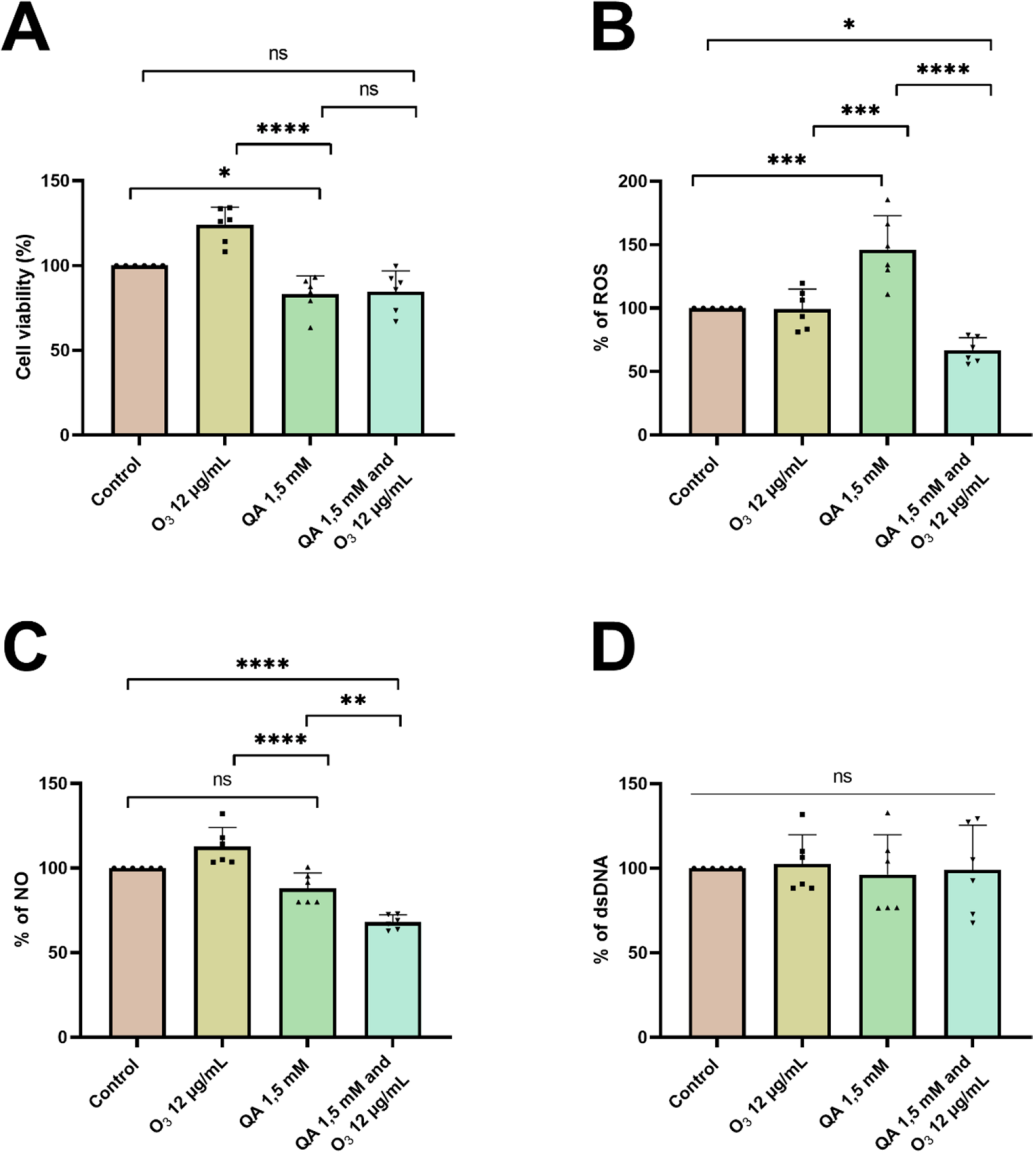
Effects of quinolinic acid and ozone on the viability of BV-2 cells (**A**), levels of reactive oxygen species (**B**), nitric oxide production (**C**), and quantification of free ds-DNA (**D**). Results are expressed as mean ± standard deviation, with individual data points shown in each parameter (six experimental replicates and are representative of at least three independent experiments; *n* = 6). Statistically significant differences were determined by two-way ANOVA with multiple comparisons between the co-treatments and indicated by asterisks (*p < 0.05; ** *p* < 0.01; *** *p* < 0.001; **** *p* < 0.0001). Statistical significance was set at *p* ≤ 0.05. QA: Quinolinic Acid; O_3_: Ozone

To investigate the effects of ozone in activated microglia, QA, a well-known toxic metabolite of the kynurenine pathway, was selected for its ability to experimentally induce neurotoxicity. Analysis of cell viability (MTT) showed that exposure to the QA significantly reduced cell viability to 83% compared to the control (*p* = 0.032). This reduction supports the cytotoxic effects of QA. The co-treatment group was not statistically different from the QA group alone or the control group (*p* > 0.05), suggesting that O_3_ did not provide significant protection against the QA-induced reduction in viability (Fig. 3A).

Exposure to QA led to a substantial increase in ROS levels, approximately 46% above baseline (*p* = 0.0005). This finding strongly supports the role of QA in inducing oxidative stress. However, when O_3_ was co-administered with QA, a notable reduction was observed, decreasing to 66.7 ± 9.9% relative to the QA-exposed group (*p* < 0.0001). This suggests a potential antioxidant or scavenging effect of O_3_ in mitigating QA-induced oxidative stress (Fig. 3B). In addition, NO production showed no significant change following QA when compared to the control (*p* > 0.05). However, the co-treatment (QA + O_3_) significantly reduces NO levels (68 ± 4.2%; *p* = 0.001) when compared to the control group and the QA group alone (*p* < 0.05; Fig. 3C). No significant changes in ds-DNA levels were observed under all experimental conditions (*p* > 0.05; Fig. 3D).

### Molecular O_3_ Effects in a Stress Environment

#### Apoptosis and Necrosis

**Fig. 4:**
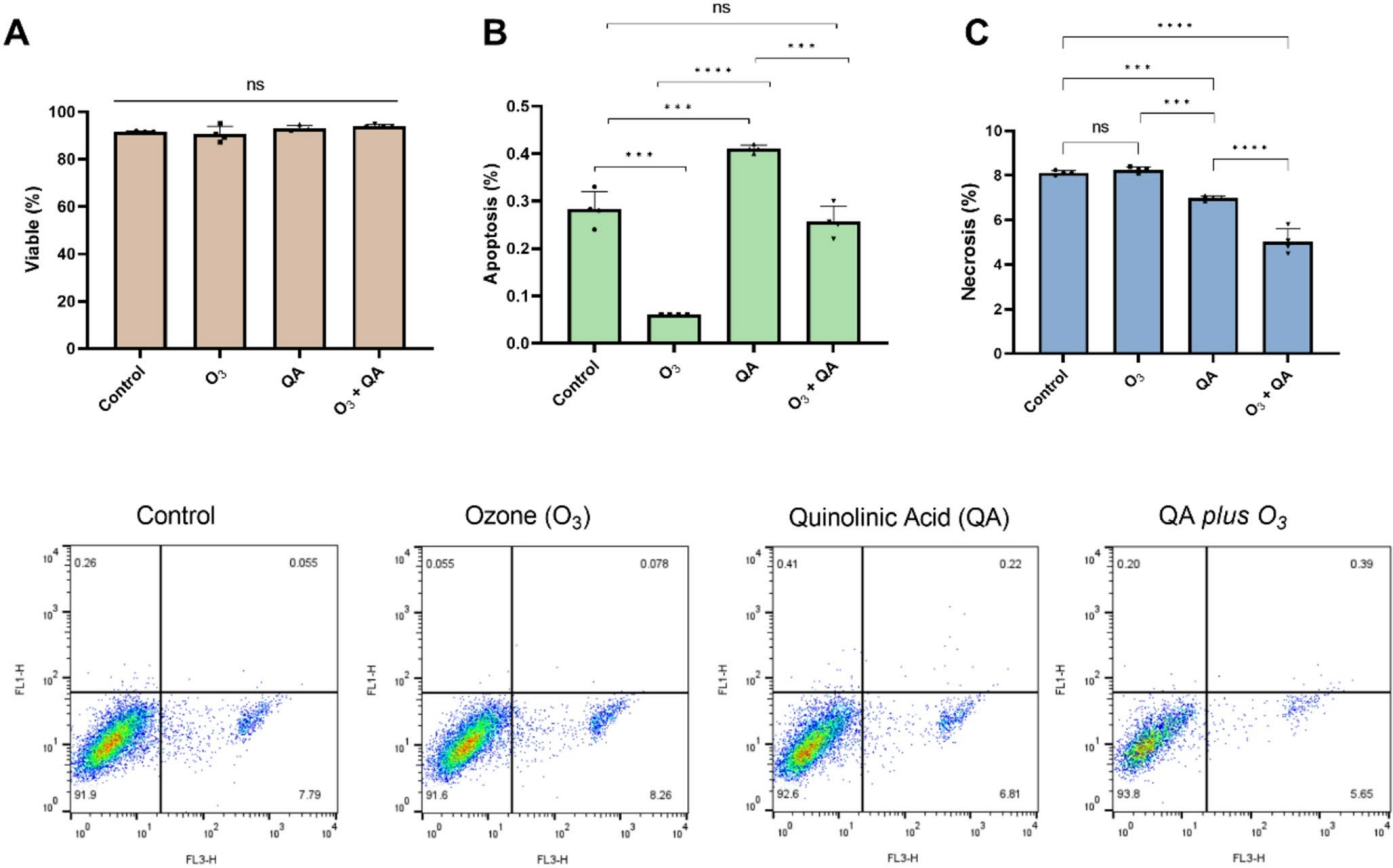
The effects of quinolinic acid and ozone on the viable fraction (**A**), apoptosis (**B**), and necrosis (**C**) in BV-2 cells, determined by flow cytometry using the Annexin V-FITC/Propidium Iodide (PI) assay. Results are expressed as mean ± standard deviation, with individual data points shown in each parameter (four experimental replicates and are representative of at least two independent experiments; *n* = 4). Statistically significant differences were determined by two-way ANOVA with multiple comparisons between the co-treatments and indicated by asterisks (*p < 0.05; ** *p* < 0.01; *** *p* < 0.001; **** *p* < 0.0001). Statistical significance was set at *p* ≤ 0.05. QA: Quinolinic Acid; O_3_: Ozone

Flow Cytometry analysis, a precise and robust technique, quantified the populations of viable, apoptotic, and necrotic cells within the experimental groups. In Fig. 4A, no impacts on viable cell fraction were revealed following exposure to the various agents, whether administered individually or in combination (*p* > 0.05). However, the apoptotic fraction exhibited strong divergences. A highly significant decrease in apoptosis was noted in the groups treated exclusively with O_3_ (0.06 ± 0.001%) when compared to the control group (0.28 ± 0.03%; *p* < 0.001). Conversely, a distinct and significant increase in apoptotic fraction was evident in the group treated exclusively with QA (0.41 ± 0.008%; *p* < 0.001) when compared to the control group. This finding highlights the pro-apoptotic effects of QA. When O_3_ was combined with QA, the co-treatment (0.25 ± 0.03%) significantly reduced the apoptotic fraction compared to the QA-alone group (*p* < 0.0001), bringing the level back to baseline (no significant difference from control; *p* > 0.05; Fig. 4B).

Additionally. significant divergences were noted in the fraction of necrotic cells across the different treatment groups. Treatment with QA alone resulted in reducing necrosis, (6.98 ± 0.10%) compared to the control group, (8.11 ± 0.09%; *p* < 0.0008). Notably, the co-treatment further decreased necrosis, (5.03 ± 0.55%) compared to both the control group, (*p* < 0.0001) and the QA-alone group, (*p* < 0.0001). This reduction suggests a protective or mitigating effect of the combined agents against this specific cell death pathway (Fig. 4C).

#### Mitochondrial Membrane Potential (ΔΨm)

**Fig. 5:**
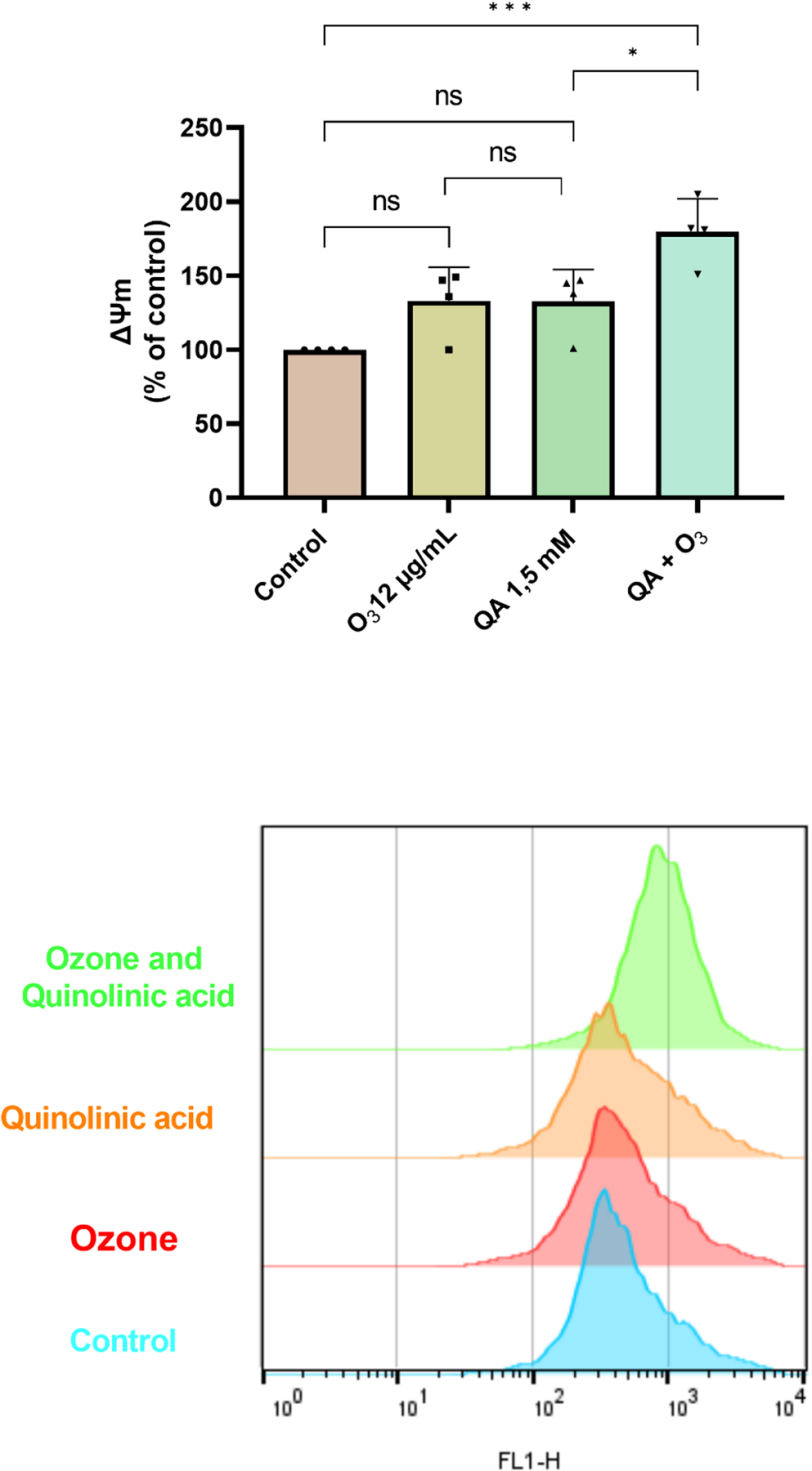
Detection of mitochondrial membrane potential (ΔΨm) in BV-2 cells treated with quinolinic acid and ozone was conducted using flow cytometry. Results are expressed as mean ± standard deviation, with individual data points shown in each parameter (four experimental replicates and are representative of at least two independent experiments; *n* = 4). Statistically significant differences were determined by two-way ANOVA with multiple comparisons between the co-treatments and indicated by asterisks (*p < 0.05; ** *p* < 0.01; *** *p* < 0.001; **** *p* < 0.0001). Statistical significance was set at *p* ≤ 0.05. QA: Quinolinic Acid; O_3_: Ozone

Cells exposed only to O_3_ maintained ΔΨm values (132 ± 12%) that were not statistically distinguishable from those of the untreated control group (*p* > 0.05). This suggests that O_3_ did not induce a significant disruption in the electrochemical gradient across the inner mitochondrial membrane, a crucial component for ATP synthesis. Similarly, cells treated exclusively with QA also exhibited ΔΨm values (132 ± 11%) that were not significantly different from the control group (*p* > 0.05). Moreover, a substantial increase was observed when O_3_ and QA were administered concurrently (178 ± 3.0%) compared to both the control group (*p* < 0.001) and the QA-alone group (*p* < 0.05). This significant elevation in ΔΨm strongly suggests a synergistic interaction between O_3_ and QA (Fig. 5).

#### Biochemical O_3_ Effects in a Stress Environment

**Fig. 6:**
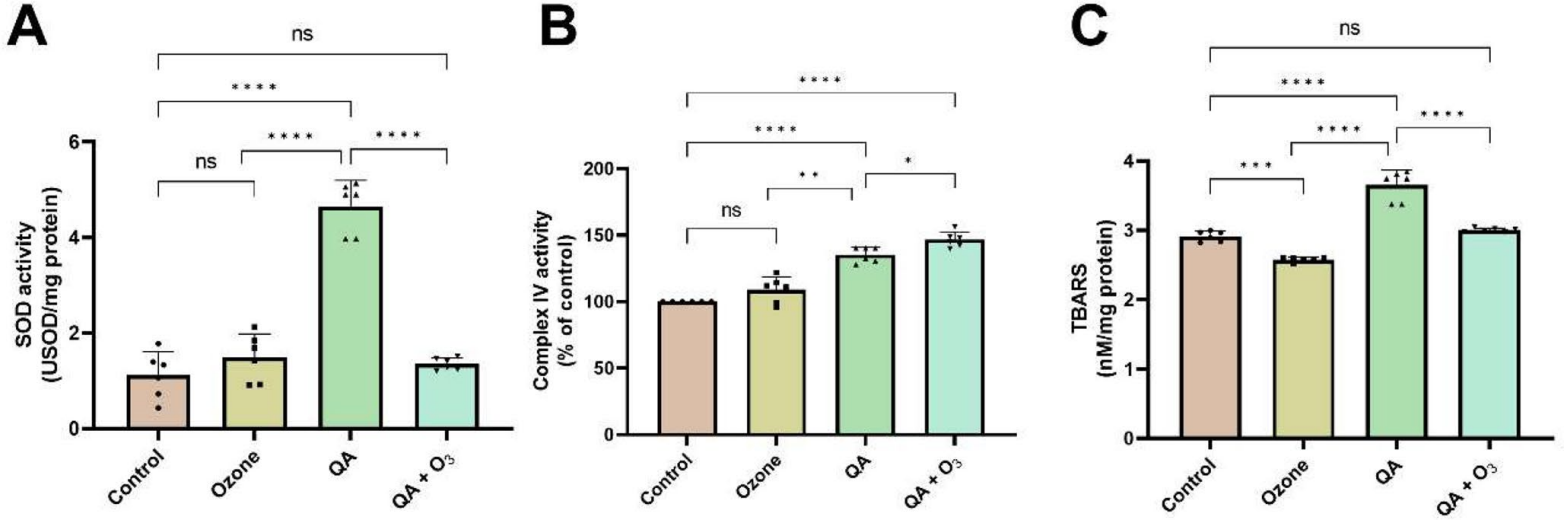
Quantification of superoxide dismutase activity (**A**), Complex IV of the electron transport chain (**B**), and TBARS levels (**C**) in BV-2 cells treated with quinolinic acid and ozone. Results are expressed as mean ± standard deviation, with individual data points shown in each parameter (six experimental replicates and are representative of at least three independent experiments; *n* = 6). Statistically significant differences were determined by two-way ANOVA with multiple comparisons between the co-treatments and indicated by asterisks (*p < 0.05; ** *p* < 0.01; *** *p* < 0.001; **** *p* < 0.0001). Statistical significance was set at *p* ≤ 0.05. QA: Quinolinic Acid; O_3_: Ozone

Activity of Superoxide Dismutase (SOD) enzyme, a critical antioxidant defense, indicated that exposure to QA alone resulted in a substantial increase, reaching 4.65 ± 0.53 USOD/mg protein (*p* < 0.0001) *versus* the control group. On the other hand, when O_3_ was combined with QA, enzymatic activity decreased significantly to 1.35 ± 0.12 USOD/mg protein (*p* < 0.0001 *versu*s QA alone). The combined treatment effectively restored SOD levels, as the QA + O_3_ group was not statistically different from the control group (*p* > 0.05). This complex interaction suggests a potential antagonistic or modulating effect of ozone on QA’s pro-oxidant defense-stimulating properties (Fig. 6A).

Furthermore, the activity of Complex IV of ETC, also known as the Cytochrome *c* Oxidase (COX) enzyme (Fig. 6B), was assessed. As for SOD findings, a significant increase in COX activity was observed in QA-treated cells (135 ± 5.62%; *p* < 0.0001), suggesting that QA not only boosts antioxidant defenses but also affects mitochondrial respiration, indicating a broader physiological impact. Intriguingly, and contrary to the protective effect seen in SOD, the co-treatment resulted in an even greater increase in COX activity, reaching 146 ± 5.7% (*p* < 0.0001 *versus* control).

To provide a comprehensive understanding of the cellular response, oxidative damage to lipids was quantified using the TBARS method (Fig. 6C). The results revealed that individual treatment with ozone significantly reduced TBARS levels (2.57 ± 0.03 nM/mg protein) when compared to the control group (2.91 ± 0.07 nM/mg protein; *p* = 0.0004). On the other hand, treatment with QA alone led to a significant elevation in TBARS levels, reaching 3.65 ± 0.22 nM/mg protein (*p* < 0.0001 *versus* control). In addition, co-treatment with O_3_ and QA demonstrated a significant reduction (3.00 ± 0.02 nM/mg protein) when compared to the group treated only with QA (*p* < 0.0001). However, the co-treatment was still statistically equivalent to the control group (*p* > 0.05). The data suggest a protective role of ozone in mitigating QA-induced lipid peroxidation, highlighting its potential in ameliorating oxidative damage.

**Fig. 7:**
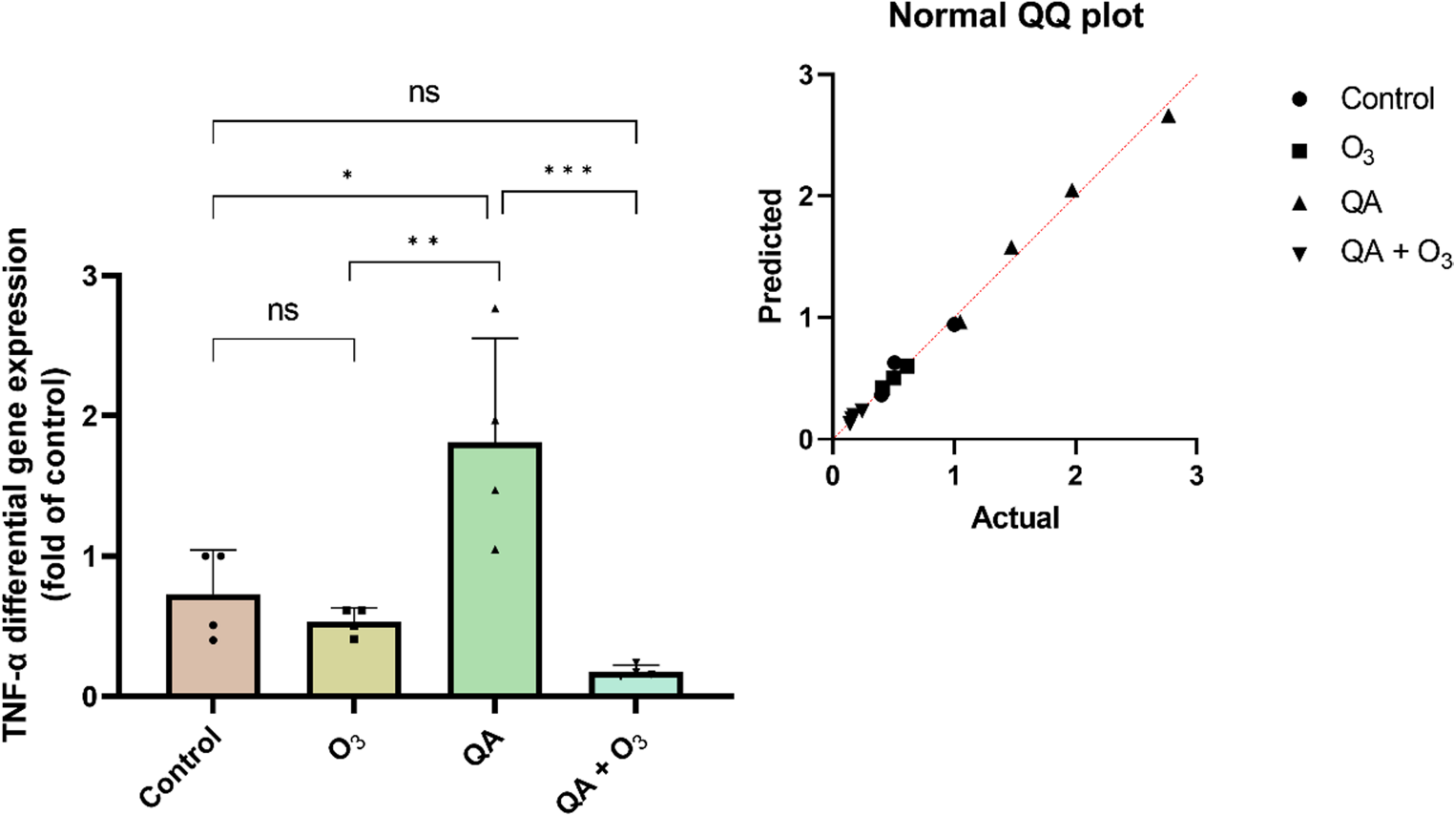
TNF-α gene expression in BV-2 cells treated with quinolinic acid, ozone, and/or both. Results are expressed as mean ± standard deviation, with individual data points shown in each parameter (four experimental replicates and are representative of at least two independent experiments; *n* = 4). Statistically significant differences were determined by two-way ANOVA with multiple comparisons between the co-treatments and indicated by asterisks (*p < 0.05; ** *p* < 0.01; *** *p* < 0.001; **** *p* < 0.0001). Statistical significance was set at *p* ≤ 0.05. QA: Quinolinic Acid; O_3_: Ozone

To investigate the impact of treatments on the inflammatory response, TNF-α gene expression levels were evaluated by qRT-PCR. As shown in Fig. 7, exposure of cells to quinolinic acid (QA) alone (1.814 ± 0.738) resulted in a significant increase in gene expression when compared to the control group (0.727 ± 0.318; *p* = 0.012). Co-treatment with quinolinic acid and ozone (0.178 ± 0.044) caused a highly significant reduction in gene expression when compared to the QA-alone group (*p* = 0.0005). This reduction effectively brought TNF-α levels back to baseline, as the co-treatment group was not statistically different from the control group (*p* > 0.05). These data indicate that ozone may play a role in modulating the inflammatory response induced by quinolinic acid.

To better understand spectrophotometric and fluorometric outcomes (Table 1), it was summarized the effects of individual exposure to ozone and quinolinic acid, as well as the synergistic or antagonistic effects observed during co-treatment conditions.

**Table 1:** Summary of biochemical and molecular effects observed in BV-2 cells exposed to optimal concentration of ozone (O_3_; 12 µg/mL) and quinolinic acid (QA; 1.5 mM) for 24 h

		Spectrophotometry Assay							Flow Cytometry Assay			RT-PCR
		MTT	ROS	NO	dsDNA	Complex IV	SOD	TBARS	Apoptosis	Necrosis	ΔΨm	TNF-α
Compared to the control	O_3_	↑	ND	ND	ND	ND	ND	↓	↓	ND	ND	ND
	QA	↓	↑	ND	ND	↑	↑	↑	↑	↓	ND	↑
	O_3_ *plus* QA	ND	↓	↓	ND	↑	ND	ND	ND	↓	↑	ND
Compared to the QA	O_3_	↑	↓	↑	ND	↓	↓	↓	↓	↑	ND	↓
	O_3_ *plus* QA	ND	↓	↓	ND	↑	↓	↓	↓	↓	↑	↓

Legend: MTT: cell viability; dsDNA: double-stranded DNA; NO: nitric oxide; ROS: reactive oxygen species; RT-PCR: Reverse Transcription Polymerase Chain Reaction; TNF-α: Tumor Necrosis Factor alpha; SOD: Superoxide dismutase; ΔΨm: mitochondrial membrane potential; TBARS: Thiobarbituric acid reactive substances; ND: no statistical differences; ↑: increase; ↓: decrease

## Discussion

In recent years, the use of ozone has grown significantly in the treatment of various health conditions (Sciorsci et al. 2020). Notable examples include fibromyalgia, treated via autohemotherapy (Tirelli et al. 2019); dental disinfection with ozonated water mouthwash (Sen and Sen 2020); and lumbar disc herniation, treated with ozone gas injection (Migliorini et al. 2020). Among the different forms of application, autohemotherapy stands out for its broad spectrum of uses, ranging from circulatory disorders and viral diseases to certain types of cancer (Bocci 2010). However, the effects of ozone in neurodegenerative diseases (NDs) are still poorly understood and remain a promising field for investigation.

In this study, low to high ozone concentrations were evaluated in murine BV-2 microglial cells, both in the presence and absence of quinolinic acid (QA), a tryptophan catabolite implicated in NDs. Initially, safe ozone concentrations were determined. An observed increase in cell viability was noted at low and moderate concentrations (5–12 µg/mL; Fig. 2A), without concomitant alterations in reactive oxygen species levels (Fig. 2B). This finding is consistent with the concept of hormesis, where the low doses act as a beneficial trigger, acting cellular defenses. We hypothesize that this positive concentration window primes the cells by activating redox-sensitive transcription factors, such Nrf2 (Cisterna et al. 2020), thereby enhancing the endogenous antioxidant capacity prior to QA insult. Comparable findings have been documented, indicating that low to moderate ozone concentrations (3–10 µg/mL) do not compromise viability in human fibroblasts (Santos et al. 2021), human keratinocytes (Valacchi et al. 2016), murine adipocytes (Cisterna et al. 2020), and neuroblastoma (Orlandin et al. 2022) cell lines. These investigations further corroborated our current observations. Conversely, exposure to elevated ozone concentrations (> 40 µg/mL) has been shown to induce cellular damage, as previously reported by Lacavalla et al. (2022), in microglial cells.

It is known that ozone can induce the formation of 4-hydroxynonenal (HNE) (Valacchi et al. 2016), an endogenous lipid peroxidation product that plays a critical role in apoptosis pathways and inflammasome activation (Hsu et al. 2022). In a previous study, an increase in HNE levels was observed in mouse lungs after ozone inhalation (Fakhrzadeh et al. 2004). Recent research suggests that HNE, peroxynitrite, and nitric oxide (NO) may be involved in apoptotic processes (Andrabi et al. 2023). In line with these findings, this study’s data indicate that in microglial cells treated with ozone at the lower concentrations, levels of NO, a crucial inflammation marker, remain unchanged, suggesting it was not able to induce nitrosative stress in microglial cells.

Fakhrzadeh et al. (2004) reported that macrophages isolated from ozone-exposed mice produced increased amounts of NO and peroxynitrite. Similar results were observed by Pendino et al. (1996), who exposed rats to ozone and isolated alveolar macrophages, verifying an inflammatory response mediated by these markers. In the study by Pierini and Bryan (2015), NO levels varied with time, increasing between 24 and 48 h after ozone exposure, declining after 72 h, and returning to basal levels. In our study, NO levels remained statistically unchanged in the therapeutic range of O_3_ (0 to 20 µg/mL) compared to the control group (Fig. 2C). However, a concentration-dependent trends towards increased NO was noted at the highest concentrations (40 and 70 µg/mL), though this increase was not statically significant when compared directly to the control group. This suggests that while high ozone concentrations may induce a specific NO-mediated inflammatory tendency, the optimal dose range does not induce nitrosative stress in microglial cells. Importantly, these NO findings were not accompanied by alterations in ROS release or in the amount of free dsDNA under our experimental conditions, which are stronger markers associated with cellular damage and the signaling of damage-associated molecular patterns (DAMPs).

Microglia are the primary immune cells of the brain and can recognize DAMPs. Upon recognition, these cells become activated, which can intensify inflammation and oxidative stress. Microglial activation can also affect tryptophan metabolism, leading to increased production of kynurenine, a product of this pathway associated with various neurodegenerative and neuropsychiatric diseases (Beurel et al. 2020; Visentin et al. 2025). Kynurenine targets NMDA receptors, leading to neurotoxic effects (Chen et al. 2021; Hestad et al. 2022). In this study, we observed a decrease in glial viability when cells were exposed to 1.5 mM of QA, corroborating other studies that reported its cytotoxic effect in CNS cell cultures and co-cultures (Feng et al. 2017; Pierozan et al. 2018; Santos et al. 2025; Rigotti et al. 2025). In addition, Feng et al. (2017) described that QA activates microglia and increases cytokine production, mediating the cytotoxicity induced by this catabolite.

Furthermore, Ganzella et al. (2006) observed that QA induced brain lesions in rats and substantially increased ROS generation. These findings align with the data of the present study, demonstrating microglia’s elevated ROS release after 24 h of QA exposure (Fig. 3B). This suggests that cellular membrane lipid peroxidation promotes ROS release into the extracellular environment. This hypothesis is corroborated by TBARS assay data, which exhibited a significant increase in lipid peroxidation in the quinolinic acid-treated group (Fig. 6C). Previous studies indicate that QA is associated with increased lipid peroxidation (Santamaría et al. 1997; Št’astný et al. 2004; Verma et al. 2018). Additionally, evidence suggests that ozone can react with polyunsaturated fatty acids in cell membranes, generating lipid peroxidation byproducts such as HNE (Bocci et al. 2009). These byproducts, known as lipoperoxides, at micromolar doses, can act as messengers of oxidative stress, contributing to the activation of endogenous cellular defense mechanisms, such as the SOD enzyme. The results of our research revealed that treatment with ozone led to a significant reduction in TBARS levels compared to the control group, reinforcing the idea that O_3_ acts as a primary antioxidant modulator in basal conditions. Conversely, treatment with QA alone led to a significant elevation in TBARS levels. However, when ozone was co-administered with QA, a notable reduction in lipid peroxidation was observed, suggesting a protective effect against QA-induced damage. In fact, the co-treatment restored TBARS levels to those statistically equivalent to the control group, supporting the idea that ozone efficiently activates endogenous defense mechanisms, aiding in the control of ROS and lipid peroxidation.

In multiple sclerosis patients, ozone (20 µg/mL) demonstrated a significant improvement in the activity of antioxidant enzymes, such as SOD, responsible for neutralizing the superoxide anion radical (O_2_.^−^) (Delgado-Roche et al. 2017). In the current study, we observed that 12 µg/mL of ozone normalized SOD activity after QA-induced upregulation (Fig. 6A). The role of QA in antioxidant enzymes is not yet fully understood. Previous studies have observed an increase in SOD activity after QA exposure in the striatum of rats (Santana-Martínez et al. 2019; Ferreira et al. 2020). Conversely, a decline in SOD activity was reported in the study by Maya et al. (2018). Our findings suggest that QA may elevate O_2_.^−^ levels, thereby inducing an increase in SOD activity. However, in the presence of ozone, it appears to assist in maintaining SOD activity at basal levels. This aligns with the ozone paradox, wherein, despite exhibiting oxidizing properties, it can indirectly mediate redox balance through antioxidant defenses.

Mitochondrial O_2_.^−^ production can be partially explained by the observed results in COX enzyme activity. Cytochrome *c* oxidase (COX), located in the mitochondrial electron transport chain (ETC), is a primary site of O_2_.^−^ generation (Brand 2016). The findings of this research did not reveal significant differences in COX activity after ozone exposure compared to the control. Nevertheless, we observed a significant increase in COX activity following QA exposure, which was unexpectedly further increased by O_3_ co-treatment (Fig. 6B). Correlating with this, the QA + O_3_ group exhibited mitochondrial hyperpolarization (Fig. 5), which represents a persistent and highly significant increase in ΔΨm. This represents a bioenergetic paradox and indicates that O_3_ failed to normalize QA-induced ETC hyperactivity, suggesting that its protective mechanism does not involve the direct repair of mitochondrial metabolic dysfunction. A previous study evaluated the impact of ozone at high dose (50 µg/mL) on mitochondrial complexes, and the authors did not observe significant differences in COX activity when compared to the control group. They suggest that this result may be attributed to the complex’s adaptive capacity, enabling it to cope with stressful conditions induced by ozone exposure (Oliveira et al. 2024). Another study investigated the effects of ozone inhalation on mitochondrial complexes in hippocampal microglial cells from sedentary and active rats, finding no significant differences in COX activity among the groups (Valdez et al. 2020). We can infer that ozone’s protective effects mainly target oxidative stress and inflammation caused by ETC dysfunction, rather than fixing the core metabolic defect, indicating a non-dependent mitochondrial action mechanism.

Previous studies have reported mitochondrial membrane depolarization in response to ozone exposure, which can lead to mitochondrial dysfunction and increased ROS production (Li et al. 2021; Tian et al. 2022). However, other studies did not observe significant alterations in mitochondrial membrane permeability (ΔΨm), even highlighting ozone’s capacity to inhibit apoptosis in neuronal cells (Cai et al. 2020). These findings suggest that ozone exhibits a biphasic effect dependent on the cellular redox state, administration route, and dosage. For instance, direct contact of ozone gas with pulmonary cells can induce metabolic dysfunctions due to the lack of a robust antioxidant system in the pulmonary environment (Al-Hegelan et al. 2011; Rogers and Cismowski 2018). In contrast, in the CNS, neuroglial cells possess the capacity to provide energetic support and antioxidant protection to neurons, thereby assisting them in resisting stressors (Jessen 2004).

Tumor necrosis factor-alpha (TNF-α), one of the most potent pro-inflammatory cytokines, plays a central role in coordinating immune responses. It can promote the proliferation of various cell types or induce apoptotic signaling, depending on the cellular context and receptor engagement (Vincenzi et al. 2021). In the present study we have focused on evaluating the TNF-α gene expression using a very sensitive method since this specific cytokine plays a central role not only in terms of neuroinflammation in general, but it is directly related to the kynurenine pathway as an important metabolite, as well as QA could be a result-product against the exposure to high concentrations of TNF-α (Pemberton et al. 2009; Okamoto et al. 2024). Furthermore, it was already shown that TNF-α signaling can directly influence microglial activation and exacerbate QA-induced neuroinflammation (Feng et al. 2017), showing that these mechanisms are closely related through a cross-talking activity characterizing a two-way feedback process. Consistent with this, our data demonstrated a marked increase in TNF-α gene expression in the group treated solely with QA (Fig. 7). Additionally, our results revealed that co-treatment (QA and ozone) led to a pronounced downregulation of TNF-α gene expression. This observation suggests that ozone may mitigate QA-induced damage through multiple, converging mechanisms. One plausible pathway involves the modulation of redox balance by reducing excessive ROS; therefore, ozone could indirectly suppress TNF-α transcription via attenuation of redox-sensitive signaling cascades, particularly the NF-κB and Mitogen-Activated Protein Kinases (MAPK) pathways, both of which are critical drivers of pro-inflammatory cytokine expression. This hypothesis is consistent with prior in vivo and in vitro evidence indicating that ozone can downregulate pro-inflammatory cytokines, including TNF-α, in models of oxidative and inflammatory injury (Zamora et al. 2005; Williams et al. 2015; Güçlü et al. 2016; Lacavalla et al. 2022). The results found here corroborate previous studies, since the cellular exposure to QA was able to increase the transcription of the TNF-α gene, while ozone could negatively modulate this overexpression.

Mechanistically, elevated TNF-α may act synergistically with excessive ROS to trigger mitochondrial dysfunction, increase lipid peroxidation, and activate apoptotic cascades, as previously reported (Vincenzi et al. 2021). Therefore, to complement the biochemical findings, flow cytometry analysis was performed, aiming to provide insight into cell fate modulation. QA alone caused a significant increase in apoptotic fraction, while the co-treatment (QA + O_3_) significantly reduced apoptosis to control levels (Fig. 4B). These protective effects are supported by studies in other models: in human hepatocellular carcinoma cells, O_3_ at high doses induced apoptosis and decreased mitochondrial membrane potential (Tang et al. 2021), while in vivo models of nerve root injury, therapeutic ozone significantly decreased cleaved caspase 3 expression and alleviated apoptosis (Wu et al. 2018). Furthermore, the co-treatment exhibited the lowest necrosis values (Fig. 4C). Crucially, the significant increase in apoptosis accompanied by low necrosis levels suggests that it is actively driving the cell toward a regulated programmed death pathway, rather than uncontrolled, acute destruction. This stabilization into the apoptotic pathway is further reinforced by O_3_ action. In many pathological models, triggering a programmed death pathway leads to a compensatory decrease in the unprogrammed, necrotic pathway. It was already shown, for instance, in BV-2 microglia under neurotoxicity-induction (Sun et al. 2022), thereby supporting the proposed mechanism. QA, which can induce cytotoxicity and oxidative stress, might synergize with the adaptive response triggered by O₃, enhancing stress tolerance and further reducing necrosis, as observed in melanoma cells exposed to kynurenine’s metabolites (Basson et al. 2024). These findings suggest that O_3_ may precondition cells to better manage QA-induced toxicity, thereby reducing necrosis.

We hypothesized that the O_3_-mediated control over oxidative stress and TNF-α is sufficient to halt the QA-initiated programmed cell death, thereby achieving cellular stability and reducing both apoptosis and necrosis, despite the persistent mitochondrial anomaly (COX/ΔΨm hyperpolarization). Previous research in a renal injury model has shown that ozone inhibits apoptosis by reducing the phosphorylation of JNK and p38 MAPK (Wang et al. 2018). Similarly, studies on nerve root inflammation demonstrated that O_3_ suppresses apoptosis by blocking the NFκB signaling pathway (Wu et al. 2018).

**Fig. 8:**
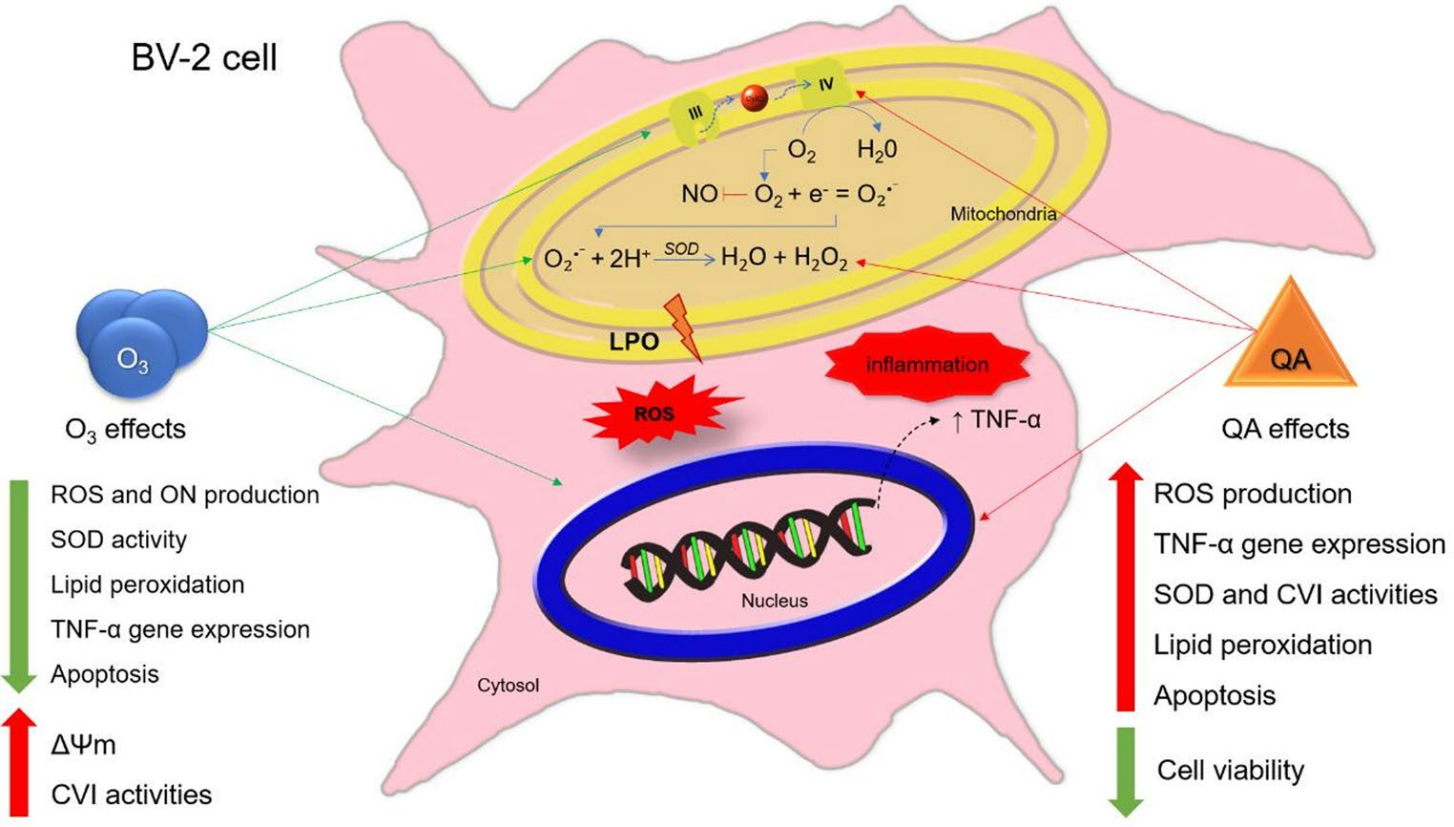
Schematic representation of the hypothesized combined mechanism of action of quinolinic acid and ozone

Considering the results regarding the impacts of treatments with ozone and/or QA, Fig. 8 presents the proposed mechanism of action of these two agents. The exposure of the BV-2 cell line to QA led to an increase in the activity of complex IV (CIV) of ETC, which resulted in a greater production of O₂•⁻ and concomitant lipid peroxidation and ΔΨm alteration. With complex IV being more active, the oxygen available to react with NO decreases, leading to a reduction in its levels. Alongside, there is an increase in ROS production. These events promote an increase in SOD activity to contain the excess superoxide radical; this also results in increased expression of the TNF-α gene. Conversely, O_3_ acts as a potent redox modulator. Even with the persistent increased CIV activity and ΔΨm, the induction of oxidative stress by QA in an ozonized microenvironment is reduced by the restoration of SOD activity, reduction of lipid peroxidation, ROS production, and TNF-α gene expression. Together, our results reinforce that ozone may act not only as an antioxidant modulator but also as a regulator of inflammatory gene expression, potentially offering therapeutic benefit in conditions characterized by excitotoxicity and neuroinflammation.

Although this study provides strong evidence of the neuroprotective potential of O_3_, we acknowledge some limitations. First, the use of the BV-2 murine immortalized cell line, while an established in vitro model, may not fully recapitulate the behavior of primary microglia or the human microglial populations. Second, the experiments are limited to a single time point (24 h), which does not capture potential longer-term or delayed effects of ozone exposure. Third, the in vitro delivery of ozonized medium differs substantially from clinical administration methods, making it difficult to directly extrapolate these results to therapeutic contexts. Despite the limitations, our findings offer valuable insights into the biological mechanisms of ozone action, which can serve as a basis for future in vivo and clinical research. Further studies using primary microglial cultures and long-term analyses are needed, however, to fully clarify the upstream effects of ozone on microglial cells.

## Conclusion

Data obtained from the in vitro safety profile indicate that ozone, at concentrations below 20 µg/mL, proved safe for microglial cells. Specifically, at a concentration of 12 µg/mL, ozone demonstrated the ability to increase cell viability without inducing oxidative or nitrosative stress, and without causing DNA damage and inflammation. Additionally, it was observed that exposure of the BV-2 microglial cell line to 1.5 mM quinolinic acid resulted in increased production of ROS, elevated apoptotic fraction, higher SOD activity and TNF-α gene expression levels, and lipid peroxidation. These effects, however, were reversed by the co-exposition of ozone, highlighting its potential as a redox state modulator. Although further research is necessary, these results provide evidence that ozone, within an optimal concentration window, can help mitigate microglial activation. Therefore, this study can contribute to the advancement of understanding brain diseases associated with oxidative stress and inflammation and opens possibilities for the development of novel therapeutic strategies. 

## Supplementary Information

Below is the link to the electronic supplementary material.

Supplementary Material 1

## Data Availability

Data will be made available on request.
