# Supplementary material for: Protective Effects of Ozone against Quinolinic Acid-Induced Redox Imbalance in Murine BV-2 Microglial Cells
Source: Neurotox Res. 2026 Apr 16;44(3):21. doi: 10.1007/s12640-026-00798-y (PMC13086755; doi:10.1007/s12640-026-00798-y)
Supplement: Supplementary file 1 — Supplementary Material 1 [file 12640_2026_798_MOESM1_ESM.docx]

**Protective effect of ozone against quinolinic acid-induced redox imbalance in murine BV-2 microglial cells**

Pedro Henrique Zatti^1^, Nicole Peyrot da Silva, Marina Rigotti^1^, Fernando Joel Scariot^2^, Carolina Bordin Davidson^3^, Alencar Kolinski Machado^3^ and Catia Santos Branco^1*^

^1^ Laboratory of Oxidative Stress and Antioxidants/Institute of Biotechnology, Universidade de Caxias do Sul (UCS), RS, Brazil. Corresponding author: [csbranc1@ucs.br](mailto:csbranc1@ucs.br)

^2^ Laboratory of Enology and Applied Microbiology/Institute of Biotechnology, Universidade de Caxias do Sul (UCS), RS, Brazil

^3^ Cell Culture and Bioactive Effects Laboratory, Universidade Franciscana (UFN), Santa Maria, RS, Brazil


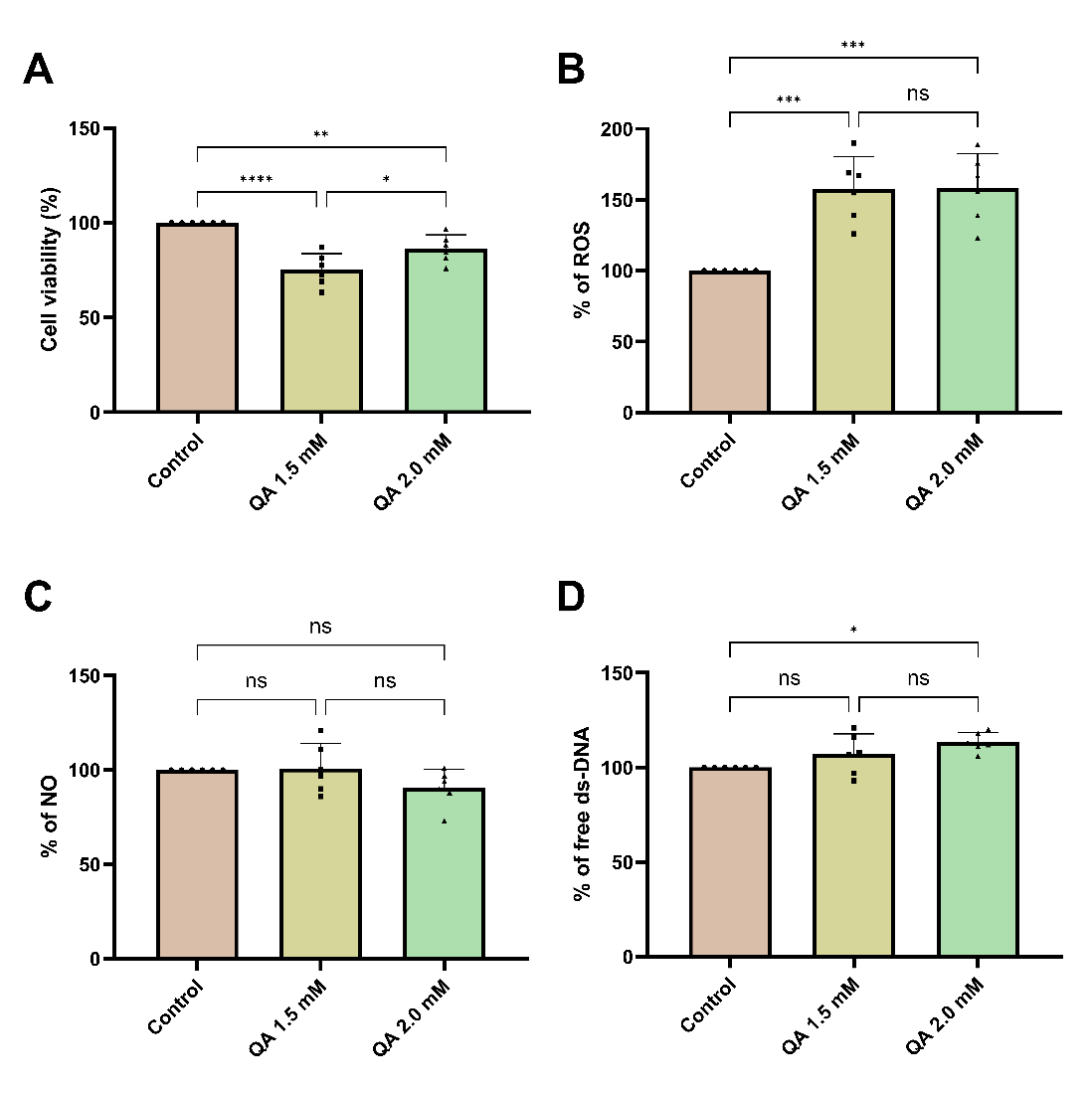


**Figure S1.** Effects of quinolinic acid (QA) after 24 hours of exposure (1.5 mM and 2.0 mM) on the viability of BV-2 cells (**A**), levels of reactive oxygen species (**B**), nitric oxide production (**C**), and quantification of free ds-DNA (**D**). Results are expressed as mean ± standard deviation (SD), with individual data points shown in each parameter (six experimental replicates and are representative of at least three independent experiments; n=6). Statistically significant differences were determined by one-way ANOVA with comparisons between the control and the QA concentrations and indicated by asterisks (**p* <0.05; ** *p* <0.01; *** *p* <0.001; **** *p* <0.0001). Statistical significance was set at *p* ≤0.05. QA: Quinolinic Acid.
